# Proteolytic degradation and potential role of onconeural protein cdr2 in neurodegeneration

**DOI:** 10.1038/cddis.2016.151

**Published:** 2016-06-02

**Authors:** J-Y Hwang, J Lee, C-K Oh, H W Kang, I-Y Hwang, J W Um, H C Park, S Kim, J-H Shin, W-Y Park, R B Darnell, H-D Um, K C Chung, K Kim, Y J Oh

**Affiliations:** 1 Department of Systems Biology, Yonsei University College of Life Science and Biotechnology, Seoul 120-749, Korea; 2 Dominick P. Purpura Department of Neuroscience, Albert Einstein College of Medicine, New York, NY 10461, USA; 3 Graduate School of Medicine, Korea University, Ansan 425-707, Gyeonggi-do, Korea; 4 Division of Pharmacology, Department of Molecular Cell Biology, Sungkyunkwan University School of Medicine, Suwon 440-746, Gyeonggi-do, Korea; 5 Laboratory of Molecular Neuro-Oncology, Howard Hughes Medical Institute, The Rockefeller University, New York, NY 10065, USA; 6 Division of Radiation Cancer Biology, Korean Institute of Radiological & Medical Sciences, Seoul 01812, Korea; 7 Department of Brain and Cognitive Sciences, Daegu Gyeongbuk Institute of Science and Technology (DGIST), Daegu 711-873, Korea

## Abstract

Cerebellar degeneration-related protein 2 (cdr2) is expressed in the central nervous system, and its ectopic expression in tumor cells of patients with gynecological malignancies elicits immune responses by cdr2-specific autoantibodies and T lymphocytes, leading to neurological symptoms. However, little is known about the regulation and function of cdr2 in neurodegenerative diseases. Because we found that cdr2 is highly expressed in the midbrain, we investigated the role of cdr2 in experimental models of Parkinson's disease (PD). We found that cdr2 levels were significantly reduced after stereotaxic injection of 1-methyl-4-phenylpyridinium (MPP^+^) into the striatum. cdr2 levels were also decreased in the brains of post-mortem PD patients. Using primary cultures of mesencephalic neurons and MN9D cells, we confirmed that MPP^+^ reduces cdr2 in tyrosine hydroxylase-positive dopaminergic neuronal cells. The MPP^+^-induced decrease of cdr2 was primarily caused by calpain- and ubiquitin proteasome system-mediated degradation, and cotreatment with pharmacological inhibitors of these enzymes or overexpression of calcium-binding protein rendered cells less vulnerable to MPP^+^-mediated cytotoxicity. Consequently, overexpression of cdr2 rescued cells from MPP^+^-induced cytotoxicity, whereas knockdown of cdr2 accelerated toxicity. Collectively, our findings provide insights into the novel regulatory mechanism and potentially protective role of onconeural protein during dopaminergic neurodegeneration.

Cerebellar degeneration-related protein 2 (cdr2), an onconeural protein, is associated with paraneoplastic cerebellar degeneration (PCD).^1, 2, 3^ Under physiological conditions, cdr2 expression is restricted to cerebellar Purkinje neurons, brain stem neurons, and testes.^4, 5^ However, cdr2 is ectopically expressed in breast or ovarian tumors of PCD patients, resulting in the generation of autoantibodies^6, 7, 8^ that are associated with neurodegeneration of Purkinje neurons.^9, 10, 11, 12^ Although the regulation of cdr2 is not well understood, an early study suggests that cdr2 is phosphorylated by PKN,^13^ and a more recent study shows that cdr2 is ubiquitinated by anaphase-promoting complex/cyclosome (APC/C) and degraded by proteasomes during the exit from mitosis.^14^ Despite these advances, the regulatory mechanisms and potential role of cdr2 in neurodegenerative disorders have not been explored.

Parkinson's disease (PD) is a neurodegenerative disorder characterized by a selective loss of dopaminergic neurons in the substantia nigra (SN) pars compacta that is associated with both motor defects and nonmotor symptoms.^15^ Mitochondrial dysfunction, oxidative stress, and inflammation are proposed to underlie the pathogenesis of familial and sporadic forms of PD.^16, 17^ Accumulating evidence indicates that protease activation plays a critical role in the progression of neurodegeneration in PD.^18, 19, 20, 21, 22, 23, 24, 25, 26, 27^ In our previous studies, we observed the activation of caspase and calpain in neurotoxin-induced dopaminergic neurodegeneration^28, 29^ and found that degradation of endogenous substrates by activated proteases leads to neurodegeneration.^30, 31^ Therefore, in the present study, we investigated the expression and protease-mediated regulation of cdr2 in experimental models of PD. We found that cdr2 is downregulated by calpain and the ubiquitin proteasome system and that the restoration of cdr2 levels renders dopaminergic neurons less vulnerable to 1-methyl-4-phenylpyridinium (MPP^+^)-mediated cytotoxicity. To our knowledge, it is the first report providing evidence that cdr2 is proteolytically regulated and may play a neuroprotective role in drug-induced model of neurodegeneration.

## Results

### cdr2 is highly expressed in the midbrain of normal adult rats

**Figure 1:**
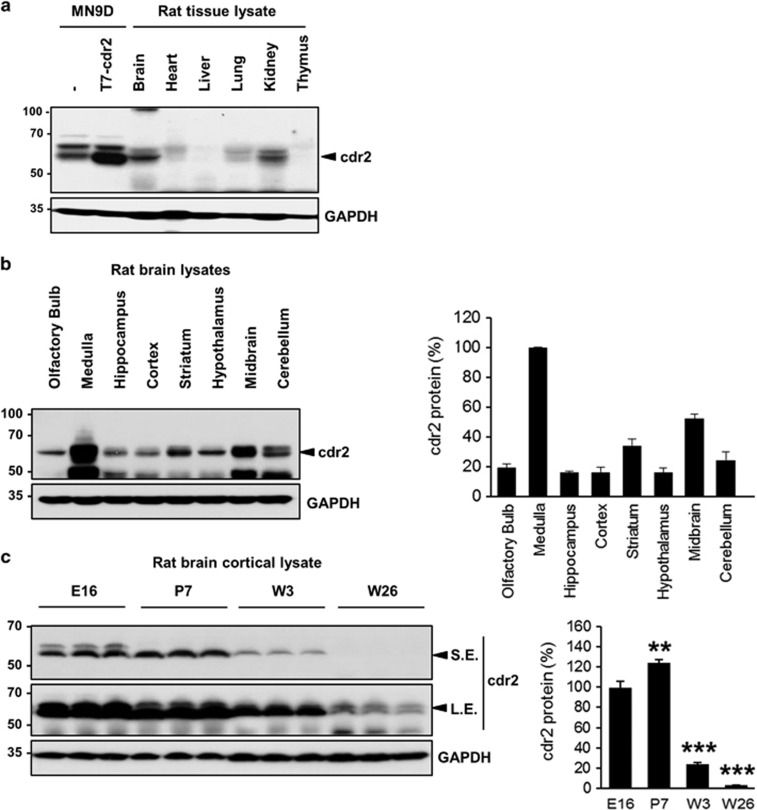
cdr2 expression in rat tissues. (**a**) cdr2 protein levels in tissue lysates (50 *μ*g) from adult rats (8 weeks old, male) were determined by immunoblotting with anti-cdr2 antibody. Lysates from MN9D dopaminergic neuronal cells transfected with or without T7-tagged mouse cdr2 were used as a positive control for the cdr2 band. Mouse monoclonal anti-GAPDH antibody was used as a loading control. (**b**) Immunoblot analysis comparing cdr2 levels across various brain regions. (**c**) Age-dependent expression of cdr2 in the cortex (E, embryonic day; P, postnatal day; W, postnatal week). Both short-term-exposed (S.E.) and long-term-exposed (L.E.) blots were presented. After normalization to GAPDH level, cdr2 levels in each lane were calculated relative to cdr2 levels in the medulla (**b**) or the E16 cortex (**c**). Data are shown as the mean±S.D. of three independent experiments. **P<0.01; ***P<0.001

Previous studies show that cdr2 is normally expressed in cerebellar Purkinje neurons but is ectopically expressed in breast and ovarian tumors of PCD patients.^4, 5, 32^ To further characterize the normal expression pattern of cdr2, lysates from various tissues from adult rats were immunoprobed with anti-cdr2 antibody. We found that cdr2 was highly expressed in the brain and kidney, whereas the heart and lung showed lower cdr2 expression (Figure 1a). This distinct spatial pattern of cdr2 expression prompted us to investigate cdr2 levels in more specific regions of the brain. We found that the medulla and midbrain showed the highest expression of cdr2, whereas the cerebellum, where Purkinje neurons reside, showed relatively lower cdr2 expression (Figure 1b). Double immunofluorescent localization of tyrosine hydroxylase (TH) and cdr2 revealed that both TH-positive and -negative cells highly expressed cdr2 in the midbrain including ventral tegmental area, SN pars compacta, and SN pars reticulata (Supplementary Figure S1). Varying levels of cdr2 were expressed in other brain regions including hippocampus, cortex, striatum, and hypothalamus (Figure 1b). In a preliminary study, quite equivalent levels of cdr2 were detected in the spinal cord and olfactory bulb (data not shown). We also found abundant cdr2 expression in the cerebral cortex of prenatal and early postnatal rats and a dramatic downregulation in adult rats (Figure 1c), suggesting the temporal regulation of cdr2 expression in the brain. Invariably, we observed more than one band of cdr2. The *in vitro* phosphatase assay showed that the upper bands represent the phosphorylated forms of cdr2 (data not shown). Although we did not pursue this observation further, it is worthy to note that a serine/threonine kinase PKN (also known as protein kinase C-related kinase 1), which is involved in the pathogenesis of Alzheimer's disease and amyotrophic lateral sclerosis,^33, 34^ interacts with and phosphorylates cdr2.^13^

### cdr2 protein is decreased in the SN of rodent models of PD and post-mortem PD patients

**Figure 2:**
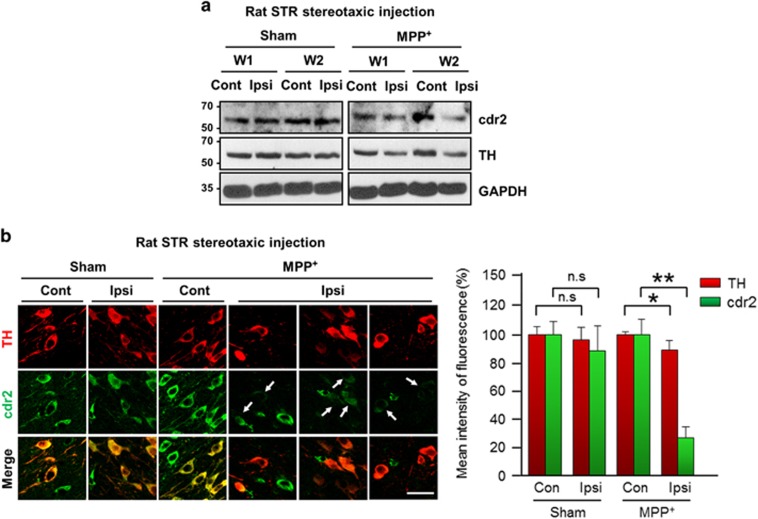
Downregulation of cdr2 in MPP^+^-injected rat brains. (**a**) Rats received a unilateral stereotaxic injection of saline (sham) or MPP^+^ (100 *μ*mol) into the striatum (STR). The indicated periods (W, week) after surgery, and cdr2 levels in the ipsilateral (Ipsi) or contralateral (Cont) SN were determined by immunoblot analysis using anti-cdr2 antibody. Mouse monoclonal anti-TH antibody was used to assess the severity of damage to dopaminergic neurons. (**b**) At 2 weeks after stereotaxic injection of MPP^+^ into the STR, SN sections were immunolabeled with anti-TH antibody and human PCD patient CSF followed by incubation with fluorescence-tagged secondary antibodies. Arrows indicate cdr2 staining in TH-positive neurons after MPP^+^ treatment. Scale bar: 40 *μ*m. The mean intensity of TH and cdr2 fluorescence in the Ipsi side was measured and expressed as a percentage of that in the sham-treated Cont side. Data were obtained from five randomly selected microscopic fields per section, with 16–20 sections per rat (~8–10 neurons per section). Data are shown as the mean±S.D. of 4–5 rats. *P<0.05; **P<0.01; n.s., not significant

**Figure 3:**
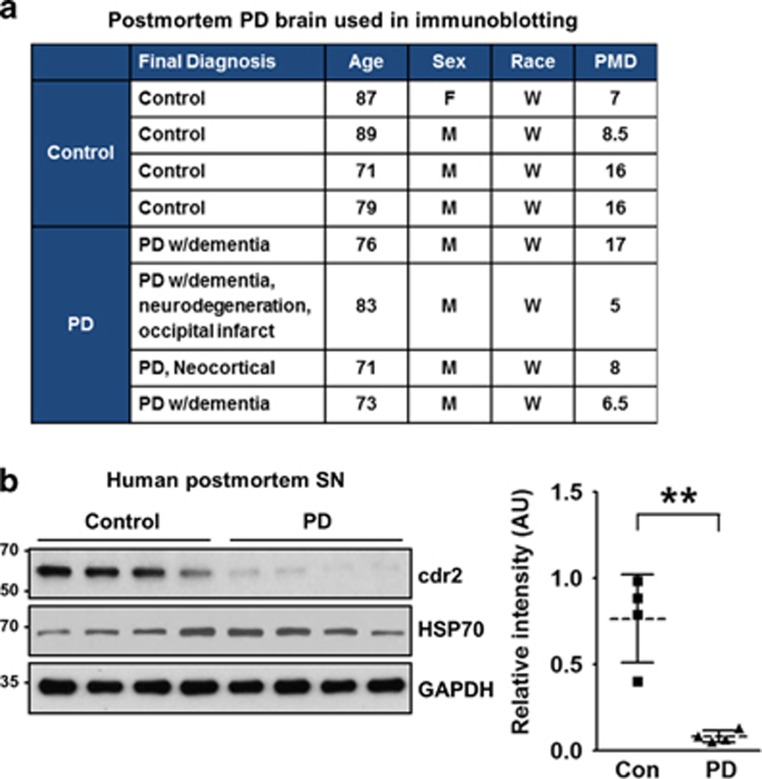
Downregulation of cdr2 in brains from post-mortem PD patients. (**a**) Characteristics of PD patients and age-matched controls, including diagnosis, age, sex, race, and post-mortem delay (PMD; h). (**b**) Tissue lysates from the SN of post-mortem humans were subjected to immunoblot analysis using anti-cdr2 antibody and anti-HSP70 antibody. After normalization against GAPDH, relative intensity of cdr2 in brains from post-mortem PD patients or age-matched controls. Data are shown as mean±S.D. **P<0.01

PD is characterized by a selective loss of dopaminergic neurons in the SN and subsequent dopamine deprivation in the striatum. Our finding that cdr2 is highly expressed in the midbrain led us to examine the regulation of cdr2 in PD pathogenesis. First, we measured levels of cdr2 protein using rodent models of PD established by stereotaxic unilateral injection of MPP^+^ (100 *μ*mol) into the striatum. In the ipsilateral side of the SN pars compacta injected with MPP^+^ for 2 weeks, the total number of TH-positive dopaminergic neurons was reduced by ~29% over the sham control group or the contralateral side of MPP^+^ injection, as determined by unbiased stereological cell counts (Supplementary Figure S2a). At 1 or 2 weeks after MPP^+^ injection into the striatum, cdr2 levels were reduced in the ipsilateral side of the SN pars compacta (Figure 2a and Supplementary Figure S2b). No discernible decrease of cdr2 was detected in the contralateral side of the midbrain or in sham controls. Any obvious decrease of cdr2 was not observed in the SN pars reticulata (Supplementary Figure S2b). Similar pattern of cdr2 reduction in the ipsilateral side of the midbrain was also observed in rats that received stereotaxic injection of MPP^+^ into the middle forebrain bundle, another rodent model of PD (data not shown). This MPP^+^-induced decrease in cdr2 levels was associated with a reduction of TH protein, indicating that decrease in cdr2 levels may be ascribed to a loss of TH-positive dopaminergic neurons or lower expression levels of cdr2 in dying dopaminergic neurons or both. Therefore, we next determined whether cdr2 levels are decreased in individual TH-positive dying neurons in the SN. At 2 weeks after MPP^+^ injection, cdr2 levels were well preserved in the cytosol of TH-positive neurons in sham controls or the contralateral side of injection (Figure 2b). However, TH-positive neurons in the ipsilateral side showed morphology typical of retracted neurites, and their cdr2 levels were markedly decreased (Figure 2b, arrows). Quantitative analysis of fluorescence intensity showed a significant decrease in cdr2 levels in ipsilateral individual TH-positive neurons (Figure 2b, right). When we measured cdr2 levels in the SN of post-mortem PD patients and age-matched controls (Figure 3a), we interestingly observed a significant decrease in cdr2 levels in all four PD brains, whereas levels of HSP70, a molecular chaperone, were not altered (Figure 3b).

### MPP^+^-induced reduction of cdr2 occurs in the TH-positive dopaminergic neurons

**Figure 4:**
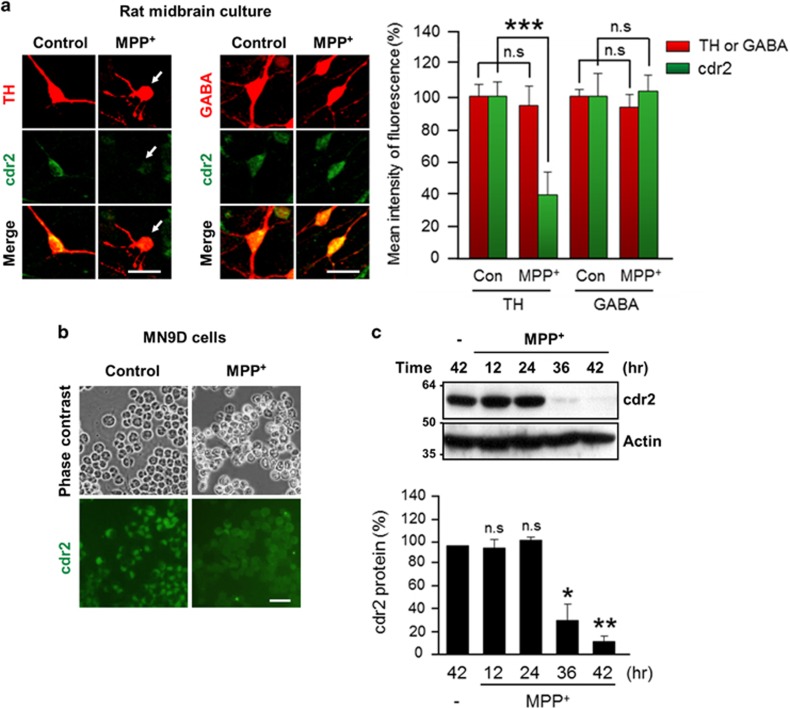
Downregulation of cdr2 in MPP^+^-treated dopaminergic neurons. (**a**) Primary cultures of rat mesencephalic dopaminergic neurons at DIV 5 or 6 were treated with or without 3 *μ*M MPP^+^ for 36 h. Cells were subjected to double immunofluorescent staining using human PCD patient CSF and anti-TH antibody or anti-GABA antibody. TH-positive neurons having retracted or fragmented neurites are indicated by white arrows. Scale bars: 20 *μ*m. Mean intensity of TH/GABA or cdr2 fluorescence was measured and expressed as a percentage of that in sham-treated control cultures. Data were obtained from 300–400 neurons from 5 to 10 randomly selected microscopic fields. Data are shown as the mean±S.D. of three independent experiments. ***P<0.001; n.s., not significant. (**b**) MN9D dopaminergic neuronal cells exposed to 50 *μ*M MPP^+^ for 36 h were immunostained using human PCD patient CSF. Phase-contrast and fluorescent images were taken using an Axiovert 100. Scale bar: 50 *μ*m. (**c**) Lysates (50 *μ*g) from MN9D cells exposed to 50 *μ*M MPP^+^ for the indicated time period were subjected to immunoblot analysis using anti-cdr2 antibody. Anti-actin antibody was used as a loading control. After normalization against actin, levels of cdr2 were expressed as a percentage of that in untreated control cells. Data are shown as the mean±S.D. of three independent experiments. *P<0.05; **P<0.01; n.s., not significant

To further determine whether the MPP^+^-induced decrease of cdr2 occurs in TH-positive dying dopaminergic cells, we used primary cultures of dopaminergic neurons derived from the rat embryonic mesencephalon or the MN9D dopaminergic neuronal cell line. Exposure of primary cultures of mesencephalic neurons to 3 *μ*M MPP^+^ for 36 h induced morphological changes such as neurite retraction and fragmentation (Figure 4a). In this condition, quantitative analysis indicated that cdr2 levels were significantly reduced in TH-positive dying neurons following MPP^+^ treatment (Figure 4a, right panel). In contrast, there were no discernible changes of cdr2 levels in *γ*-aminobutyric acid (GABA)-positive neurons in the same culture. These data along with our finding that cdr2 is preserved in neurons of SN pars reticulata suggest that the MPP^+^-induced decrease in cdr2 levels is cell-type specific. The MN9D cell line, a fusion product of mesencephalic dopaminergic neurons and N18TG neuroblastoma, was previously demonstrated to synthesize, release, and take up dopamine.^35, 36^ As determined by immunocytochemistry, exposure of MN9D dopaminergic cells to 50 *μ*M MPP^+^ for 36 h or longer caused a significant reduction in cdr2 levels, whereas numbers of cells in culture remained the same regardless of drug treatment (Figure 4b). Immunoblot analysis indicated that MPP^+^ causes a time-dependent reduction of cdr2 (Figure 4c), Taken together, our data indicated that decrease in cdr2 occurs in dying TH-positive neurons and is not simply due to MPP^+^-induced loss of dopaminergic neurons.

### Activated calpain and the ubiquitin proteasome system are responsible for MPP^+^-induced decrease of cdr2

**Figure 5:**
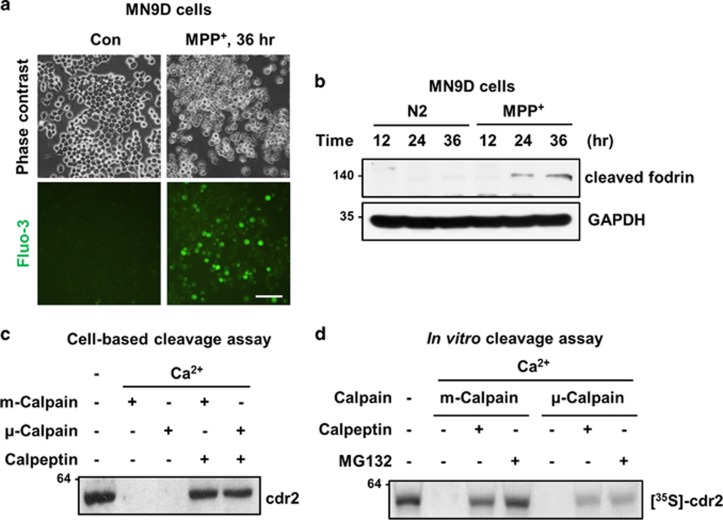
Degradation of cdr2 by calpain. (**a**) MN9D cells were treated with 50 *μ*M MPP^+^ for 36 h. Cells were then loaded with Fluo-3/AM dye and examined under a fluorescence microscope to detect levels of intracellular free Ca^2+^. Scale bar: 100 *μ*m. (**b**) Lysates (50 *μ*g) from MN9D cells treated with or without 50 *μ*M MPP^+^ for the indicated time period were subject to immunoblot analysis using monoclonal anti-fodrin antibody recognizing calpain-cleaved band. (**c**) Lysates from MN9D cells (50 *μ*g) were incubated with recombinant m-calpain (0.343 units) or *μ*-calpain (0.134 units) in the presence of 1 mM CaCl_2_. Calpeptin (50 *μ*M) was added to the lysates to block calpain activity. After the reaction, immunoblot analysis using anti-cdr2 antibody was performed to detect remaining cdr2. (**d**) For *in vitro* cleavage assay, [^35^S]-labeled cdr2 was incubated with m-calpain (0.343 units) or *μ*-calpain (0.134 units) in the presence of 1 mM CaCl_2_. Calpeptin (50 *μ*M) or MG132 (10 *μ*M) was added to the reaction mixtures. After incubation, all samples were separated by SDS-PAGE and subjected to autoradiography

Having established that cdr2 is downregulated in the brains of experimental models of PD as well as post-mortem PD patients, we next investigated the mechanism underlying MPP^+^-induced reduction of cdr2. MPP^+^-induced cell death is accompanied by or results from a burst of intracellular free Ca^2+^, leading to activation of calpain, a Ca^2+^-dependent cysteine protease in MN9D cells as previously demonstrated by us.^30, 37, 38^ Therefore, we determined whether Ca^2+^-mediated calpain activation is responsible for MPP^+^-induced reduction of cdr2. As shown in Figure 5a, many MN9D cells stained positive for fluo-3, a Ca^2+^-sensitive fluorescent dye, 36 h after 50 *μ*M MPP^+^ treatment. Quantitative analysis revealed an approximately fourfold increase in the intensity of fluo-3-stained MN9D cells relative to untreated control cells 36 h after MPP^+^ treatment (data not shown). Moreover, fodrin, a general calpain substrate, was cleaved in a time-dependent manner (Figure 5b), indicating that a rise of intracellular free Ca^2+^ leads to calpain activation in MPP^+^-treated MN9D cells. We next specifically inquired whether cdr2 is a substrate of calpain by incubating lysates of MN9D cells in calpain-activating conditions. We found that the addition of either m- or *μ*-calpain caused complete degradation of endogenous cdr2 (Figure 5c), and this event was blocked in the presence of calpeptin, a cell-permeable calpain inhibitor. To demonstrate that cdr2 is a direct substrate of activated calpain, we performed a calpain cleavage assay using *in vitro* translated [^35^S]-labeled cdr2. Both m- and *μ*-calpain led to complete degradation of cdr2 (Figure 5d) that was inhibited in the presence of calpeptin or MG132, another calpain inhibitor.^39^

**Figure 6:**
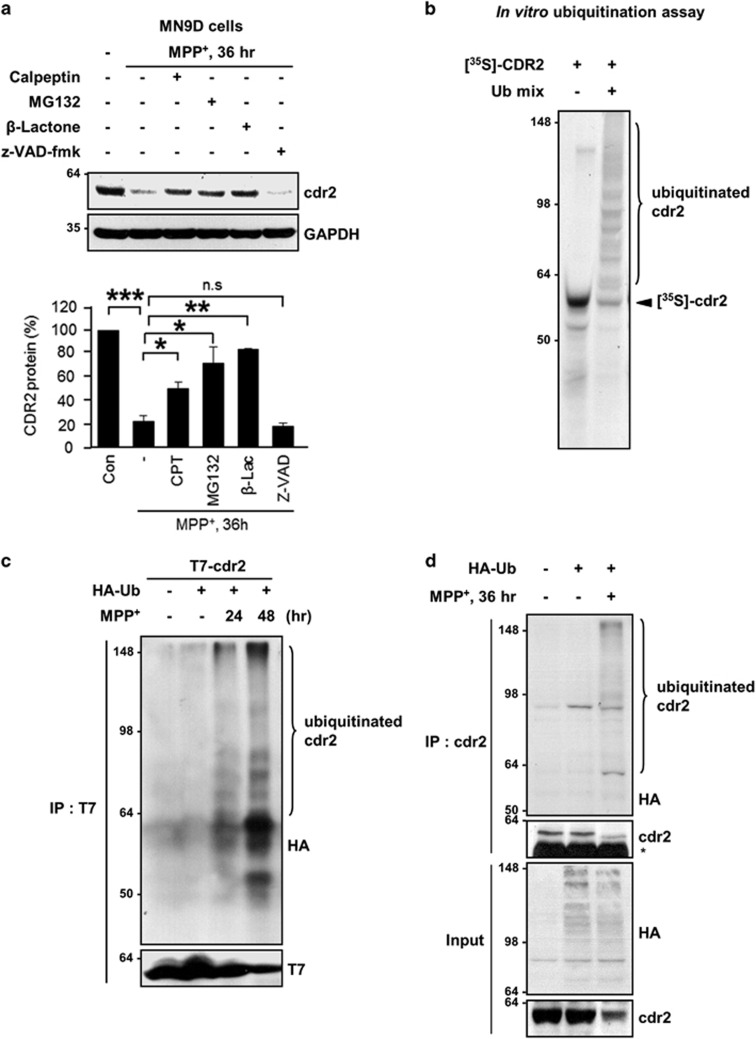
Degradation of cdr2 by the ubiquitin proteasome system. (**a**) MN9D cells were treated with 50 *μ*M MPP^+^ for 36 h in the presence or absence of calpeptin (50 *μ*M), MG132 (2.5 *μ*M), clasto-lactacystin *β*-lactone (2.5 *μ*M), or Z-VAD-fmk (100 *μ*M). Levels of cdr2 were measured by immunoblot analysis using anti-cdr2 antibody. After normalization against GAPDH, levels of cdr2 were expressed as a percentage of that in untreated controls. Data are shown as the mean±S.D. of three independent experiments. *P<0.05; **P<0.01; ***P<0.001; n.s., not significant. (**b**) [^35^S]-labeled cdr2 was incubated with or without a mixture of conjugation enzymes (E1, E2, and E3) plus ubiquitin and ubiquitin-aldehyde. Reaction mixtures were separated by SDS-PAGE and subjected to autoradiography. (**c**) MN9D cells transiently transfected with T7-tagged cdr2 plus HA-Ub were exposed to 50 *μ*M MPP^+^ for the indicated time period. To prevent proteasome-mediated degradation of cdr2, MG132 (2.5 *μ*M) was added to each culture 6 h before harvest. Cell lysates were processed for immunoprecipitation with mouse monoclonal anti-T7 and immunoblot analysis using mouse monoclonal anti-HA to detect poly-ubiquitinated cdr2. (**d**) To detect endogenous cdr2 ubiquitination, MN9D cells transiently transfected with HA-Ub were treated with or without 50 *μ*M MPP^+^ for 36 h in the presence of MG132 (2.5 *μ*M). Cell lysates were subjected to immunoprecipitation with anti-cdr2 antibody and followed by immunoblot analysis using mouse monoclonal anti-HA

We next sought to investigate whether other proteases are involved in MPP^+^-induced degradation of cdr2. MN9D cells were treated with 50 *μ*M MPP^+^ in the presence or absence of various protease inhibitors. Again, MPP^+^-induced degradation of cdr2 was significantly inhibited in the presence of calpeptin or MG132 (Figure 6a). Because MG132 can inhibit different types of proteases, including calpain, serine proteases, and proteasomes, we used a more specific irreversible proteasome inhibitor, clasto-lactacystin *β*-lactone. Cotreatment of MN9D cells with clasto-lactacystin *β*-lactone significantly blocked MPP^+^-induced degradation of cdr2, whereas cotreatment with z-VAD, a pan-caspase inhibitor, had no effect (Figure 6a). To directly determine whether cdr2 is also a substrate for the ubiquitin proteasome system, we performed *in vitro* ubiquitination assay using [^35^S]-labeled cdr2 protein. We found that poly-ubiquitinated bands of cdr2 appeared in a reaction mixture containing E1, E2, and E3 enzymes (Figure 6b). To test whether MPP^+^ induces the ubiquitination of cdr2, MN9D cells transiently transfected with T7-tagged cdr2 and HA-tagged ubiquitin (HA-Ub) were exposed to 50 *μ*M MPP^+^. Immunoprecipitation and immunoblot assay revealed that the poly-ubiquitinated cdr2 first emerged at 24 h and further increased 48 h after MPP^+^ exposure, whereas no discernible ubiquitination bands were found in untreated control cells (Figure 6c). Consistently, endogenous cdr2 was also ubiquitinated in MN9D cells transiently transfected with HA-Ub after exposure to 50 *μ*M MPP^+^ for 36 h (Figure 6d). Taken together, our results indicate that MPP^+^-induced degradation of cdr2 is mediated by at least two independent mechanisms: calpain and the ubiquitin proteasome system.

### cdr2 protects dopaminergic cells from MPP^+^-induced cell death

**Figure 7:**
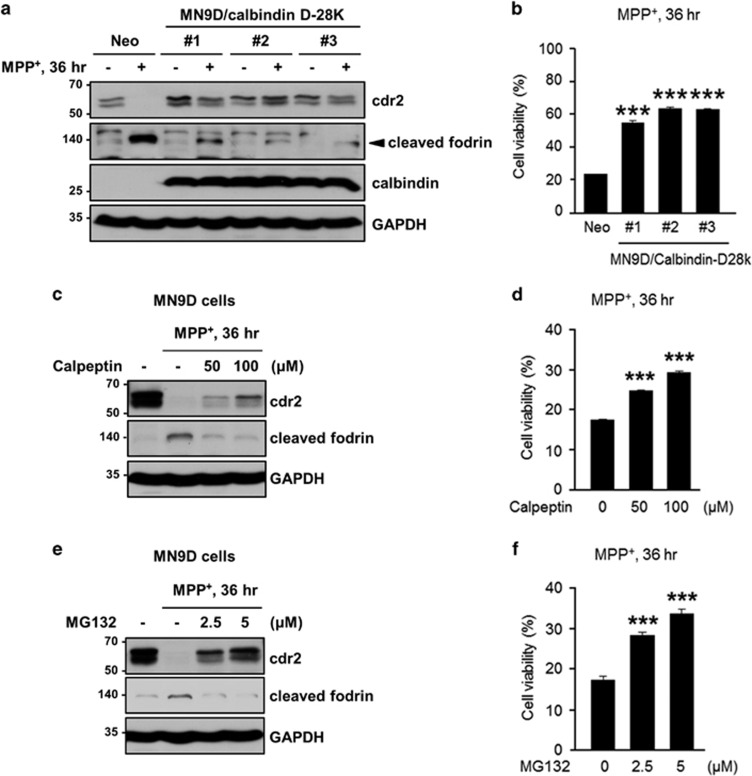
Preservation of cdr2 levels is linked to less vulnerability to MPP^+^-induced toxicity. (**a**) MN9D cells stably transfected with calbindin-D-28K (MN9D/calbindin-D-28K #1, #2, or #3) or control vector (MN9D/Neo) were exposed to 50 *μ*M MPP^+^ for 36 h. Cell lysates from the indicated clones were processed for immunoblot analysis using the indicated antibodies. Mouse monoclonal anti-calbindin-D-28K antibody was used to detect its expression in stably established clones. (**b**) Cell viability was measured by MTT reduction assay. Data are shown as the mean±S.D. of three independent experiments. ***P<0.001. (**c–f**) MN9D/Neo cells were treated with 50 *μ*M MPP^+^ for 36 h in the presence or absence of the indicated concentration of calpeptin (**c** and **d**) or MG132 (**e** and **f**). (**c** and **e**) Immunoblot analysis was performed using the indicated antibodies. (**b**, **d**, and **f**) Cell viability was expressed as a percentage of that for untreated control cells. Data are shown as the mean±S.D. of three independent experiments. ***P<0.001

**Figure 8:**
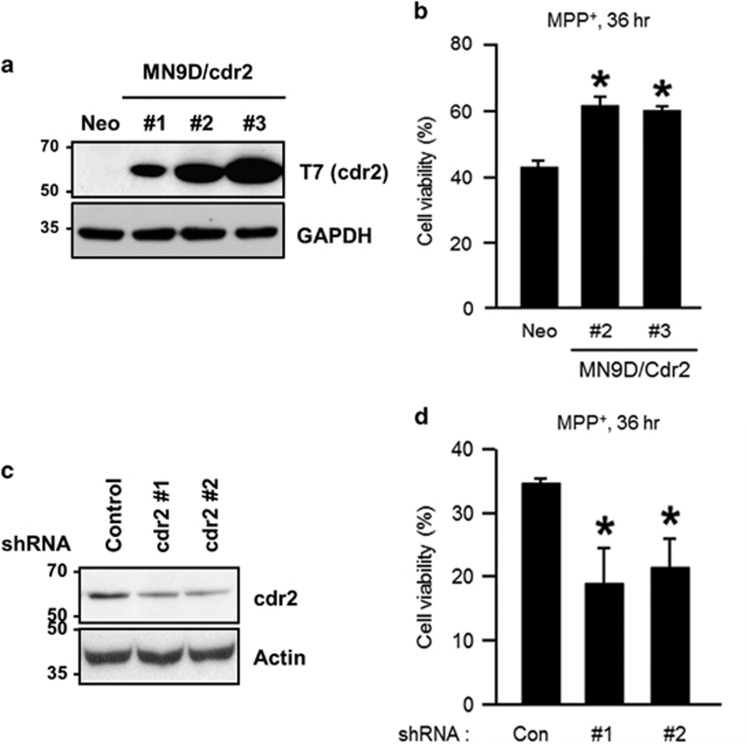
Protective role for cdr2 in MPP^+^-induced cell death. (**a**) MN9D cells were stably transfected with T7-tagged mouse cdr2 (MN9D/cdr2 #1, #2, or #3) or empty vector (MN9D/Neo). Expression levels were validated by immunoblot analysis using mouse monoclonal anti-T7 antibody. (**b**) MN9D/Neo and two highly expressing MN9D/Cdr2 cell lines were treated with 50 *μ*M MPP^+^ for 36 h. Cell viability was measured using MTT reduction assay and expressed as a percentage of that for untreated control cells. Data are shown as the mean±S.D. of three independent experiments. *P<0.05. (**c**) MN9D cells were stably transfected with cdr2 shRNA- or control shRNA-expressing vectors. Extent of cdr2 knockdown was determined by immunoblot analysis using anti-cdr2 antibody. (**d**) After treatment with 50 *μ*M MPP^+^ for 36 h, MTT reduction assay was performed. Cell viability was expressed as a percentage of that for untreated control cells. Data are shown as the mean±S.D. from three independent experiments. *P<0.05

Our observation of cdr2 downregulation in PD brain models and post-mortem human PD brains prompted us to investigate a functional role of cdr2 during dopaminergic neurodegeneration. Based on our observation that cdr2 is degraded by activated calpain following MPP^+^ treatment, we first asked whether chelation of cytosolic Ca^2+^ and/or inhibition of calpain could rescue cells from MPP^+^-mediated cytotoxicity. To test this hypothesis, we first utilized MN9D cells stably expressing calbindin-D-28K with four conserved EF hand domains and Ca^2+^ buffering activity.^40, 41^ We found that MPP^+^ (50 *μ*M)-induced decrease of cdr2 and appearance of calpain-cleaved fodrin were attenuated in all three independent MN9D/calbindin-D-28K cell lines (#1, #2, and #3) compared with a mock-transfected control cell line (MN9D/Neo; Figure 7a). Consequently, all three calbindin-expressing cell lines showed less vulnerability to 50 *μ*M MPP^+^ treatment as determined by MTT (3-(4,5-dimethylthiazol-2-yl)-2,5-diphenyltetrazolium bromide) reduction assay (Figure 7b). Similarly, pharmacological inhibition of calpain by calpeptin or MG132, which partially preserved cdr2 levels, attenuated MPP^+^-induced MN9D cell death (Figures 7c–f), suggesting that the reduced vulnerability of calbindin-expressing cells or calpeptin/MG132-treated MN9D cells may be in part attributed to preserved cdr2 levels. To directly examine whether the level of cdr2 itself affects cell survival upon exposure to 50 *μ*M MPP^+^, we established three MN9D cell lines stably overexpressing T7-tagged cdr2 and two MN9D cell lines in which cdr2 was stably silenced by transfection with short hairpin RNA (shRNA; Figures 8a and c). MTT reduction assay using two highly cdr2-expressing cell lines (MN9D/cdr2 #2 and #3) indicated that overexpression of cdr2 protects cells from MPP^+^-induced cytotoxicity (Figure 8b). In contrast, MPP^+^-induced cell death was accelerated in cdr2-silenced MN9D cells (Figure 8d). Quite similar pattern was observed when stable MN9D cells were exposed to 50 *μ*M MPP^+^ for varying time periods (Supplementary Figure S3). Interestingly, we observed that cdr2 is also decreased during 6-hydroxydopamine (6-OHDA)-induced neuronal death (Supplementary Figures S4a and b). As we previously demonstrated,^28, 29^ 6-OHDA led to reactive oxygen species-dependent MN9D cell death. As a consequence, 6-OHDA-mediated decrease in cdr2 was largely inhibited in the presence of *N*-acetyl-L-cysteine but not calpeptin. These data suggest that an additional calpain-independent degradation pathway may be involved in 6-OHDA-mediated decrease of cdr2. We also observed that 6-OHDA-induced cell death was significantly blocked in cdr2-overexpressing MN9D cells (Supplementary Figure S4c). Collectively, our results suggest that cdr2 may play a neuroprotective role in drug-induced dopaminergic neurodegeneration.

## Discussion

Although cdr2 mRNA is widely expressed, cdr2 protein expression is restricted to immune-privileged sites including the testis, brain stem, and cerebellum, suggesting that cdr2 expression is regulated by a post-transcriptional mechanism.^4, 5^ Here, we examined cdr2 expression patterns in several brain regions and found that the second most prominent site of expression was the midbrain. We also observed high levels of cdr2 expression in the striatum, a target of dopaminergic projections from the SN pars compacta. Therefore, we investigated whether cdr2 levels are altered in dopaminergic neurodegeneration. Indeed, we found lower cdr2 protein levels in the brains of post-mortem PD patients and animal PD models established by stereotaxic injection of MPP^+^ into the striatum. Using both cultured MN9D dopaminergic cells and primary cultures of mesencephalic neurons challenged with MPP^+^, we found decreased levels of cdr2 in TH-positive dying dopaminergic neurons but not in GABAergic neurons. This cell-type specificity can be addressed by the fact that GABAergic neurons is more resistant to MPP^+^ or rotenone, another PD-related drug targeting mitochondria complex I.^42^

We showed that cdr2 degradation was mediated primarily by calpain and the ubiquitin proteasome system. Consequently, pharmacological inhibition of these enzymes successfully blocked MPP^+^-induced degradation of cdr2 and subsequent dopaminergic neurodegeneration. Previous studies, including ones from our laboratory, report that MPP^+^-induced calpain activation via increased intracellular Ca^2+^ leads to cleavage of numerous cellular substrates and contributes to neuronal cell death,^28, 30, 43, 44^ supporting the notion that degradation of critical cellular proteins by activated calpain may be linked to dopaminergic neurodegeneration. An increase in intracellular free Ca^2+^ is also known to occur in acute neurodegenerative conditions such as ischemic stroke and spinal cord injury.^45, 46, 47, 48^ In preliminary studies, we found that cdr2 levels were decreased in a rodent ischemic stroke model established by middle cerebral artery occlusion and a contusion spinal cord injury model (data not shown), suggesting that cdr2 may be degraded by activated calpain during both acute and chronic neurodegeneration. Previously, it has been demonstrated that cdr2 undergoes APC/C-mediated poly-ubiquitination during the exit from mitosis.^14^ Although we did not determine the relationship between MPP^+^-induced ubiquitination of cdr2 and APC/C activity in MN9D cells, the results of our *in vitro* and cell-based ubiquitination assays raise the possibility that cdr2 is poly-ubiquitinated and subject to proteasome-mediated degradation after MPP^+^ treatment. In a separate study, we found that both Parkin and SIAH bind to cdr2 in HEK293 cells and N2 (N-2 supplement) neuroblastoma (data not shown). However, we do not yet have clear evidence of cdr2 poly-ubiquitination by either of these two E3 ligases. Therefore, further attempts are necessary to identify the specific E3 ligase for cdr2 ubiquitination.

Our previous study showed that calpain-mediated cleavage of optineurin, peripherin, or arsenical pump-driving ATPase occurs in MPP^+^-treated MN9D dopaminergic cells, and any treatments that restore their protein levels or overexpression of one of these substrates rescue MN9D cells from MPP^+^-mediated cytotoxicity.^30^ In accordance with the results of pharmacological inhibition of calpain, MN9D cells overexpressing calbindin-D-28K showed preservation of cdr2, resulting in more resistance to MPP^+^-induced cytotoxicity. Similarly, we demonstrated that MN9D cells overexpressing cdr2 are less vulnerable to MPP^+^-induced cytotoxic damage. Conversely, MPP^+^-induced cell death was accelerated in MN9D cells subjected to shRNA-mediated silencing of cdr2, suggesting that cdr2 protein levels may be positively correlated with the rate of neuroprotection in MN9D cells after MPP^+^ treatment. Therefore, we are tempting to suggest that cdr2 exerts a certain neuroprotective function and thus its degradation may be associated with drug-induced neurodegeneration. Intriguingly, we also found that shRNA-mediated knockdown of cdr2 in cultured hippocampal neurons enhanced spontaneous apoptotic cell death, raising the possibility that cdr2 may have a neuroprotective role during early development (Supplementary Figure S5). Furthermore, cdr2 morpholino-injected zebrafish embryos showed massive death of neural progenitor cells and post-mitotic differentiated neuronal cells in the spinal cord (Supplementary Figure S6). Considering that developing neurons undergo programmed cell death to control the number of neural progenitor cells and optimize neural connections between differentiating neurons and their targets in both vertebrates and invertebrates,^49^ our preliminary findings suggest that cdr2 may be a key regulator and not merely a bystander of neuronal cell survival, although it is highly speculative at present.

Previous studies by others indicate that cdr2 is primarily present in the cytoplasm and has a leucine zipper motif.^6, 13, 50, 51^ Therefore, cdr2 could bind to other proteins with a leucine zipper motif and exert transcriptional transactivation activity via binding to DNA. For example, cdr2 captures c-myc in the cytoplasm that prevents the transactivation of pro-apoptotic genes by nuclear c-myc activity in cerebellar Purkinje neurons,^10^ indicating that cdr2 can block c-myc-mediated apoptosis. As another example, cdr2 interacts with a nuclear helix-loop-helix leucine zipper protein, MRG X,^52^ and coexpression of cdr2 and MRG X prevents MRG X-induced glioblastoma cell death. The same laboratory also reports that cdr2 binds to the cell cycle-related protein MRG15 and that overexpression of cdr2 inhibits the derepression of B-myb transcriptional activity by MRG15.^53^ The B-myb transcription factor is induced in response to apoptotic stimuli, and its knockdown in neurons is protective against nerve growth factor deprivation or drug-induced cell death accompanying DNA damage.^54^ Therefore, cdr2 may serve a neuroprotective role by repressing B-myb promoter activity. Although we did not attempt to determine whether these scenarios hold true in MN9D cells, we observed that MPP^+^ treatment decreased levels of c-myc in the nucleus regardless of whether cells overexpressed cdr2 (data not shown). Therefore, it seems that a neuroprotective role of cdr2 cannot be ascribed to its known regulation of the c-myc-mediated cell death pathway, at least in MN9D cells. We also found that cdr2 has a chromosome segregation ATPase domain as determined by a database search and can bind to ATP as determined by *in vitro* ATP binding assay (data not shown). Similarly, a colocalization study performed in our laboratory indicates that cdr2 may bind to microtubules in MN9D cells (data not shown). At present, we do not know whether and how these activities of cdr2 are related to its neuroprotective role in neuronal cells. Therefore, further studies delineating the biological and neuroprotective activity of cdr2 in the nervous system are required.

In summary, we characterized the cdr2 expression profile in various regions of the brain, and examined its regulation by activated proteases and its neuroprotective function during neurotoxin-induced dopaminergic neurodegeneration. Our findings indicate that the negative regulation of cdr2 levels by activated calpain and the ubiquitin proteasome system may contribute to MPP^+^-induced neuronal cell death. Unveiling the detail mechanisms involved in regulation of cdr2 would expand our understanding of the potential role of cdr2 in pathological neurodegeneration.

## Materials and Methods

### Animals, stereotaxic surgery, and post-mortem human brains

All experimental animal procedures were in accordance with the National Institutes of Health Guide for the Care and Use of Laboratory Animals and approved by the Institutional Animal Care and Use Committees of Yonsei University. To measure tissue-specific expression of cdr2 protein, various body parts including subregions of the brain were surgically removed from Sprague-Dawley (SD) rats (Orientbio, Seongnam, Korea) at different ages. For other groups of rats, stereotaxic surgery was performed as previously described with modifications.^55^ Briefly, female SD rats (250–280 g; Daehan Biolink, Deajon, Korea) were anesthetized using an intraperitoneal injection of chloral hydrate (360 mg/kg) and placed in a stereotaxic apparatus (Kopf Instrument, Tujunga, CA, USA). Each rat received a unilateral injection of MPP^+^ (Sigma, St. Louis, MO, USA) or sterilized saline into the right striatum (100 nmol; +1 mm anteroposterior, −2.5 mm mediolateral, and -4.5 mm dorsoventral relative to bregma) according to Paxinos and Watson (1998).^56^ Injections were performed using a Hamilton syringe equipped with a 26S-gauge beveled needle driven by a syringe pump (K.D. Scientific, Holliston, MA, USA). The needle was slowly retracted 10 min after the injection. On the predetermined day after surgery, 3–5 rats in each condition were killed by CO_2_, and the SN was rapidly dissected out for immunological analysis. The SNs from four post-mortem patients with PD and four age-matched control individuals were provided by the Department of Pathology at Johns Hopkins University. Each brain underwent comprehensive neuropathological analysis.^57^

### Primary neuronal cultures

To prepare primary cultures of dopaminergic neurons, the ventral mesencephalon was removed from SD rats (Orientbio) on embryonic day (E)14 as previously described.^28^ Briefly, dopaminergic neuronal cultures were plated at 1.0 × 10^5^ cells per 1 cm^2^ ACLAR embedding film (Electron Microscopy Sciences, Fort Washington, PA, USA) precoated with 100 *μ*g/ml poly-D-lysine (Sigma) and 4 *μ*g/ml laminin (Invitrogen, San Diego, CA, USA) and maintained at 37 °C in a humidified 5% CO_2_ atmosphere in modified Eagle's medium (MEM; Gibco, Grand Island, NY, USA) supplemented with 10% fetal bovine serum (FBS; Lonza, Walkersville, MD, USA), 2 mM L-glutamine (Sigma), and 6 g/l glucose (Sigma). At 5 or 6 days *in vitro* (DIV), cultures were washed with MEM and treated with 3 *μ*M MPP^+^ for 36 h.

### MN9D cell culture, drug treatment, and cell viability

MN9D dopaminergic neuronal cultures were established from embryonic mesencephalic dopaminergic neurons by somatic fusion.^35^ As previously described by us,^44^ MN9D cells were plated on 25 *μ*g/ml poly-D-lysine precoated culture dishes or plates (Costar, Corning, NY, USA), maintained in DMEM (Gibco) supplemented with 10% FBS in an incubator with 10% CO_2_ at 37 °C, and switched to serum-free N2 medium^58^ containing various experimental reagents, including MPP^+^, calpeptin (Calbiochem, San Diego, CA, USA), MG132 (Calbiochem), *N*-benzyloxycarbonyl-Val-Ala-Asp-fluoromethylketone (Z-VAD-fmk, Enzyme Systems Products, Livermore, CA, USA), and clasto-lactacystin *β*-lactone (Calbiochem). To assess the rate of cell survival after drug treatment, MTT reduction assay was performed as previously described.^59^ Briefly, after the indicated incubation period, MTT solution was added to the culture at a final concentration of 1 mg/ml. Cells were then incubated for 1 h at 37 °C followed by lysis in 20% SDS in 50% aqueous dimethylformamide for 24 h. The optical density of dissolved formazan grains was measured at 540 nm using a microplate reader (Molecular Devices, Sunnyvale, CA, USA). Values for each treatment group were calculated as a percentage relative to the untreated control group (defined as 100% survival).

### Immunoblot analysis

The SN of post-mortem human brains was processed for immunoblot analysis as previously described by us.^31^ To measure cdr2 expression in rats, various body parts including brain regions were dissected and subjected to lysis. Briefly, dissected tissues were minced with blades, rinsed with ice-cold phosphate-buffered saline (PBS), and briefly microcentrifuged. The resulting pellets were lysed with RIPA buffer (50 mM Tris-HCl, pH 7.4, 1% NP-40, 0.25% sodium deoxycholate, 150 mM NaCl, 1 mM EDTA) containing 0.1% SDS and protease inhibitor cocktail (Roche, Mannheim, Germany) and then further homogenized with a 1-ml syringe. SN obtained from rats that received stereotaxic injection of MPP^+^ was similarly processed. For MN9D cells, cells were washed with ice-cold PBS, lysed with RIPA buffer containing protease inhibitor cocktail, and then homogenized using a 1-ml syringe. Tissue or cell lysates were microcentrifuged at 15 000 × *g* for 20 min at 4 °C. Protein content of the supernatants was measured using a Bio-Rad protein assay reagent (Hercules, CA, USA). Proteins from each sample were separated on 10–12.5% SDS-PAGE, blotted onto prewetted PVDF nitrocellulose filters (Bio-Rad), and blocked with TBST including 5% skim milk for 1 h. The blots were immunoprobed with human PCD patient serum that reacts with cdr2 (1 : 10 000, generously provided by Dr Darnell at Rockefeller University, New York, NY, USA) or rabbit polyclonal anti-cdr2 (1 : 1000; Sigma), mouse monoclonal anti-tyrosine hydroxylase (TH, a rate-limiting enzyme of dopamine biosynthesis: 1 : 1000; Pel-Freez, Rogers, AR, USA), mouse monoclonal anti-*α*-fodrin (1 : 4000; Enzo Life Science, Farmingdale, NY, USA), mouse monoclonal anti-hemagglutinin (HA; 1 : 4000; Santa Cruz Biotechnology, Dallas, TX, USA), mouse monoclonal anti-T7 (1 : 1000; Novagen, Madison, WI, USA), mouse monoclonal anti-HSP 70 (1 : 1000; Santa Cruz), or mouse monoclonal anti-calbindin-D-28K (1 : 2000; Swant, Fribourg, Switzerland). Rabbit polyclonal anti-actin (1 : 4000; Sigma) and mouse monoclonal anti-glyceraldehyde-3 phosphate dehydrogenase (GAPDH, 1 : 4000; Merck Millipore, Billerica, MA, USA) were used as loading controls.

### Immunofluorescent staining

For immunohistochemistry, rats were perfused transcardially with saline solution containing 0.5% sodium nitrate and heparin (1000 units/ml, Sigma) before fixation. Brains were fixed at 4 °C overnight with 4% paraformaldehyde in 0.1 M phosphate buffer and incubated in 30% sucrose solution for 48–72 h at 4 °C until they sank. Brains were then cut into 30-μm-thick coronal sections using a sliding microtome. Sections were processed for immunohistochemical staining for TH and cdr2. For immunocytochemistry, primary cultures of mesencephalic cells and MN9D dopaminergic cells were fixed, blocked, and incubated with primary antibodies as previously described.^28^ Primary antibodies were mouse monoclonal anti-TH (1 : 7500, Pel-Freez), rabbit polyclonal GABA antibody (1 : 200; Sigma), and human PCD patient cerebrospinal fluid (CSF, 1 : 20; generously provided by Dr Darnell at Rockefeller University) that reacts with cdr2. After extensive washes with PBS, sections or cultures were incubated at RT for 1 h with Alexa 546-conjugated goat anti-mouse antibody or Alexa 546-conjugated goat anti-rabbit antibody in combination with Alexa 488-conjugated goat anti-human antibody (1 : 200, Molecular Probes, Eugene, OR, USA). After extensive washes, sections or cultures were then mounted with Vectashield mounting medium (Vector Laboratories, Burlingame, CA, USA) and examined under a Axiovert 100 microscope equipped with an epifluorescence and digital image analyzer (Carl Zeiss, Zena, Germany) or an LSM 510 Meta Laser Scanning Microscope (Carl Zeiss). Fluorescence intensity was measured and analyzed using ImageJ software (NIH, Bethesda, MD, USA).

### Measurement of cytosolic free Ca^2+^ by Fluo-3

A method for visualization of intracellular free Ca^2+^ levels was applied to MN9D cells using Fluo-3 calcium indicator (Molecular Probes). Briefly, MN9D cells treated with or without 50 *μ*M MPP^+^ for the indicated time were loaded with 4 *μ*M Fluo-3 AM and incubated at 37 °C for 30 min. After incubation, cells were washed twice with N2-supplemented medium and examined under an Axiovert 100 microscope equipped with an epifluorescence and digital image analyzer (Carl Zeiss) at an excitation wavelength of 488 nm.

### *In vitro* and cell-based calpain cleavage assay

For *in vitro* calpain cleavage assays, the vector encoding T7-tagged mouse cdr2 in pcDNA3 was transcribed and translated in the presence of [^35^S]-methionine (Perkin Elmer, Boston, MA, USA) using a TnT Quick coupled transcription/translation system (Promega, Madison, WI, USA) according to the manufacturer's recommendations. For cell-based cleavage assay, MN9D cells were lysed in buffer containing 50 mM Tris-HCl, pH 8.0, 2 mM EDTA, and 1% Triton X-100 buffer without protease inhibitor cocktail. [^35^S]-cdr2 or cell lysates (50 *μ*g) were incubated for 1 h at 30 °C in a calpain activation buffer containing 1 mM CaCl_2_ in the presence or absence of purified m-calpain (0.343 units) or *μ*-calpain (0.134 units; both from Calbiochem) as recommended by the manufacturer. If necessary, calpeptin (50 *μ*M) or MG132 (2.5 *μ*M) was added to the reaction mixtures. Reactions were terminated by the addition of 5 × protein sample buffer followed by boiling for 5 min. The resulting products were separated on 10% SDS-PAGE gel and processed for autoradiography or immunoblot analysis.

### *In vitro* and cell-based ubiquitination assay

By using an ubiquitin protein conjugating kit (Calbiochem), *in vitro* ubiquitination of [^35^S]-cdr2 was performed as recommended by the manufacturer. Briefly, all components mixed at a total volume of 25 *μ*l were incubated for 3 h at 37 °C. The reaction was terminated by adding 5 × protein sample buffer and boiling for 5 min. The reaction mixtures were separated on 8% SDS-PAGE gel and processed for autoradiography. To assess the ubiquitination pattern of exogenous and endogenous cdr2, MN9D cells were transiently transfected with or without T7-tagged cdr2 in combination with HA-Ub and exposed to 50 *μ*M MPP^+^ for the indicated period of time. To inhibit proteasome-mediated degradation of the ubiquitinated proteins, 2.5 *μ*M MG132 was added to the culture medium. Cells were then harvested and subjected to lysis in RIPA buffer containing protease inhibitor cocktail. For immunoprecipitation, lysates (500 *μ*g) were precleared with protein A agarose (Upstate Biotechnology, Lake Placid, NY, USA) for 2 h and further incubated with either mouse monoclonal anti-T7 antibody or rabbit polyclonal anti-cdr2 antibody (Sigma) with gentle rotation overnight at 4 °C. Immunocomplexes were collected by incubation with protein A agarose for 2 h at 4 °C and subjected to centrifugation at 3000 × *g* at 4 °C for 2 min. After washing the beads three times, proteins were eluted by boiling with 1 × protein sample buffer and then separated on SDS-PAGE gel and subjected to immunoblot analysis using mouse monoclonal anti-ubiquitin antibody (Santa Cruz).

### Plasmids and transfection

T7-tagged full-length mouse cdr2 (GenBank Accession Number U88588) in pcDNA3 eukaryotic expression vector was generously provided from Dr Darnell at Rockefeller University. For constructs expressing cdr2-specific shRNA, the sequences 5′-GGATCCCGTTGATGCAACTAAATATCTCCTTGATATCCGGGAGATATTTAGTTGCATCAATTTTTTCCAAAAGCTT-3′ (#1) or 5′-GGATCCCGTTTCGCATGCTGCTCATTCATTTGATATCCGATGAATGAGCAGCATGCGAAATTTTTTCCAAAAGCTT-3′ (#2) were chosen based on recommendations by Genscript's shRNA design center (http://www.genscript.com/design_center.html) and ligated into pRNAT-U6.1/Neo vectors. GFP signal under control of the CMV promoter in the vectors was used to track transfection efficiency. MN9D cells were transfected with vector containing T7-tagged cdr2 or cdr2 shRNAs using Lipofectamine 2000 (Invitrogen) as recommended by the manufacturer. The transfected cells were maintained in the presence of 500 *μ*g/ml G418 (AG Scientific Inc., San Diego, CA, USA) for an additional 2 weeks and expanded in culture medium containing 250 *μ*g/ml G418 for further experiments.

### Statistical analyses

Data are shown as mean±S.D. Group differences were analyzed using Student's *t*-tests (unpaired, two tailed) or one-way analysis of variance (ANOVA) followed by Tukey's *post hoc* tests using GraphPad Prism 6 software (La Jolla, CA, USA). Statistical significance was set at *P*<0.05.

## Abbreviations

PD: Parkinson's disease
cdr2: cerebellar degeneration-related protein 2
APC/C: anaphase-promoting complex/cyclosome
N2: N-2 supplement
MPP^+^: 1-methyl-4-phenylpyridinium
MTT: 3-(4,5-dimethylthiazol-2-yl)-2,5-diphenyltetrazolium bromide
PCD: paraneoplastic cerebellar degeneration
TH: tyrosine hydroxylase
GABA: *γ*-aminobutyric acid
Ub: ubiquitin
SN: substantia nigra
CSF: cerebrospinal fluid
Z-VAD-fmk: *N*-benzyloxycarbonyl-Val-Ala-Asp-fluoromethylketone
*β*-lactone: clasto-lactacystin *β*-lactone
