# Supplementary figures and images for: Proteolytic degradation and potential role of onconeural protein cdr2 in neurodegeneration
Source: Cell Death Dis. 2016 Jun 2;7(6):e2240–. doi: 10.1038/cddis.2016.151 (PMC5143381; doi:10.1038/cddis.2016.151)

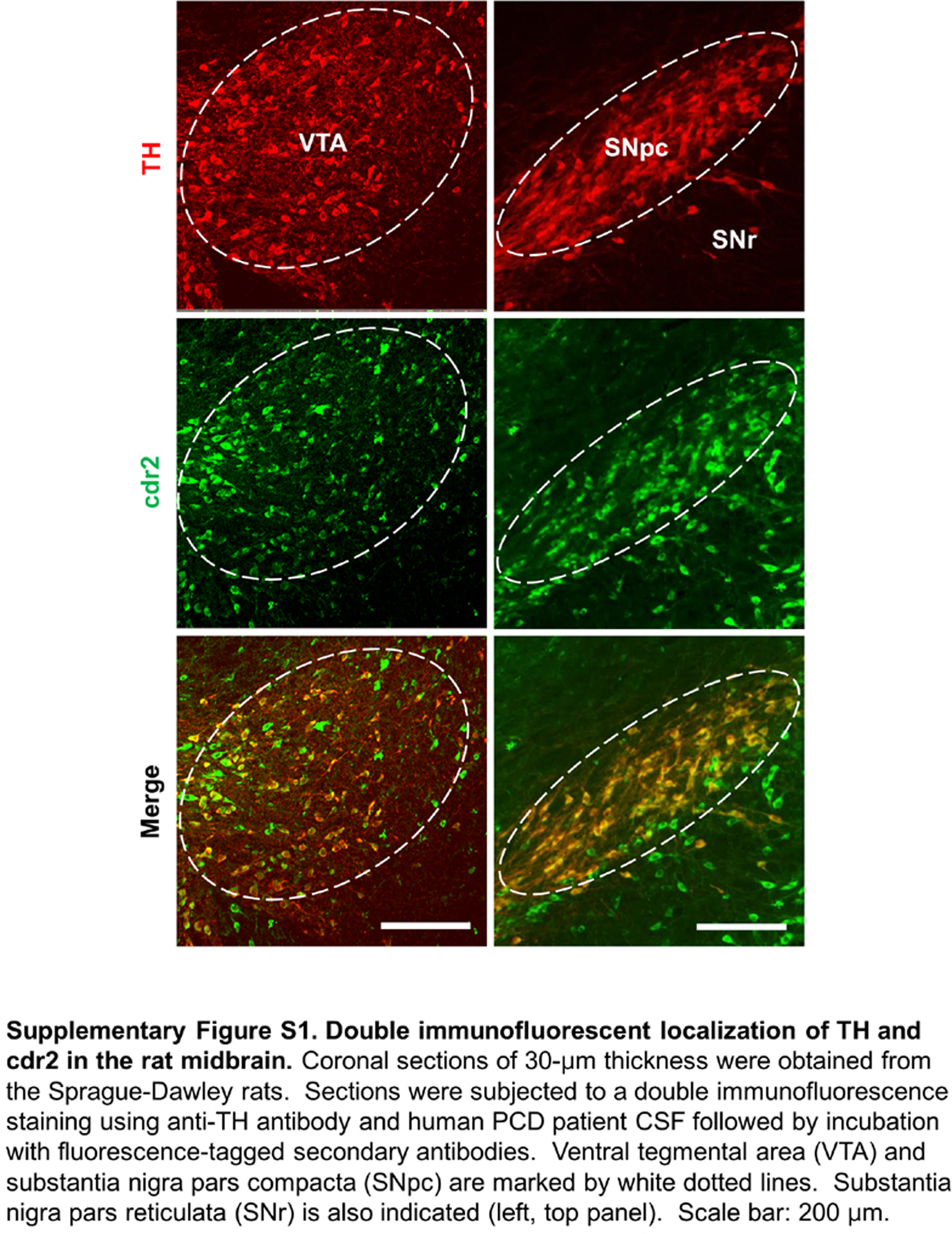

Supplement: Supplementary Figure 1 [file cddis2016151x1.tif]

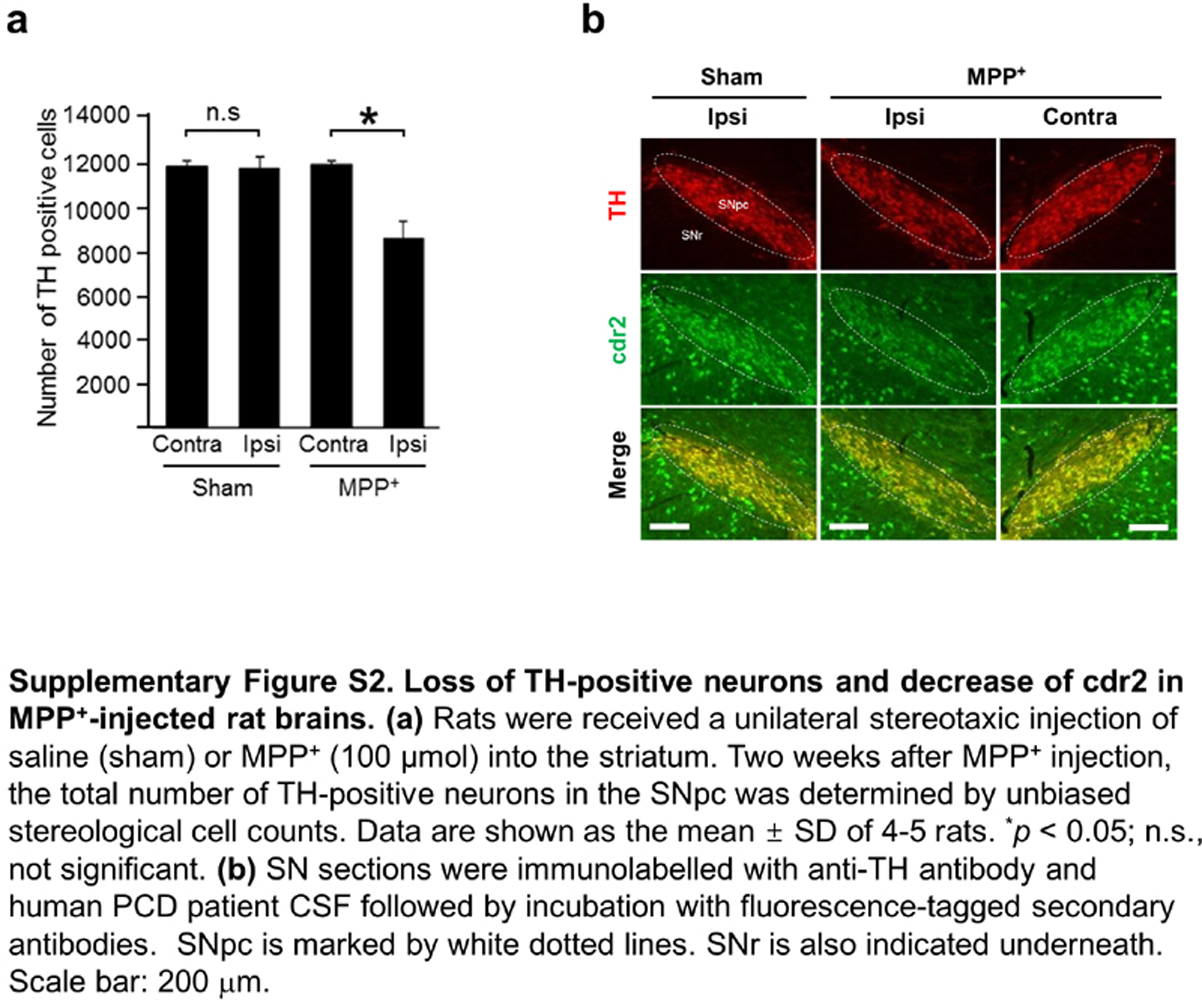

Supplement: Supplementary Figure 2 [file cddis2016151x2.tif]

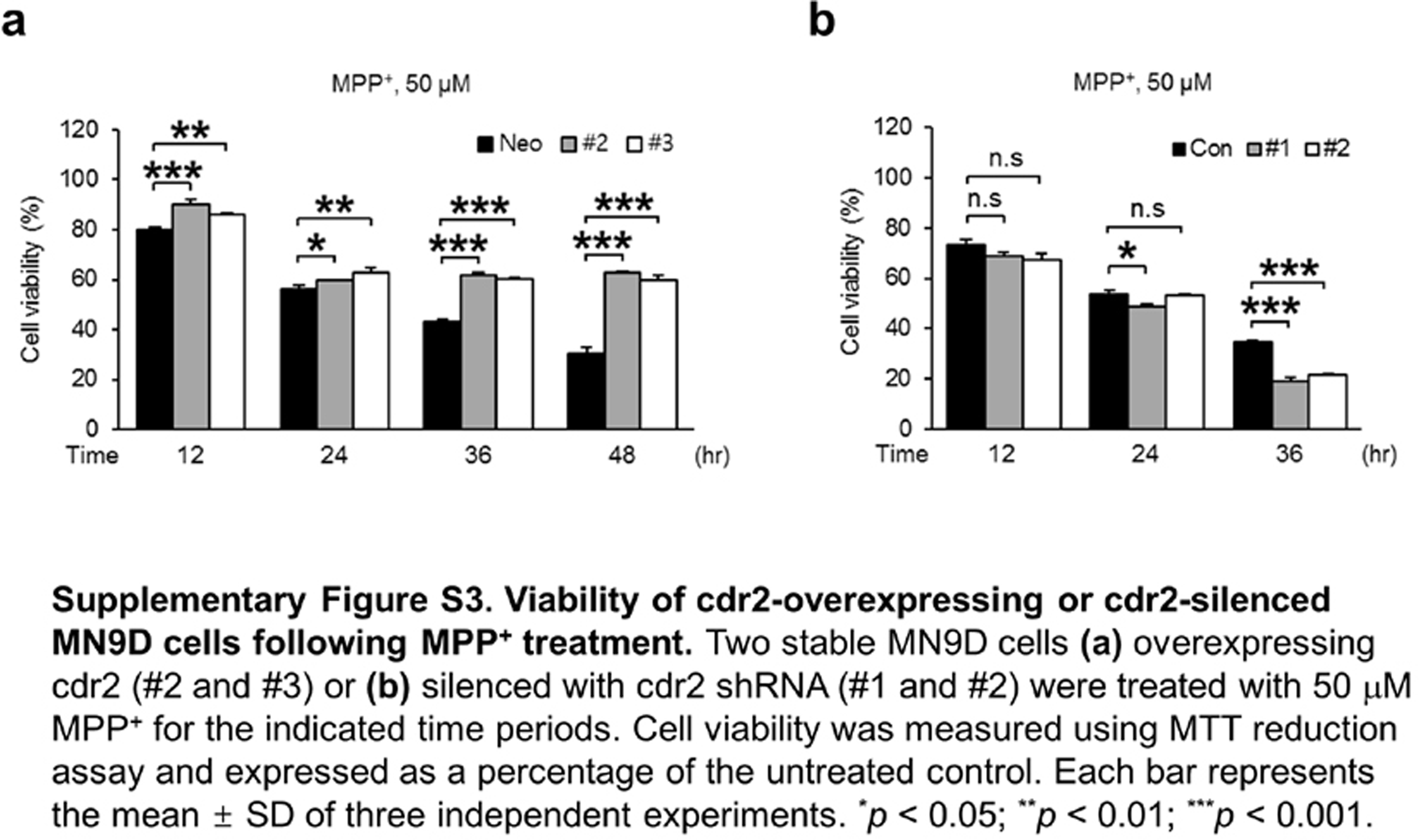

Supplement: Supplementary Figure 3 [file cddis2016151x3.tif]

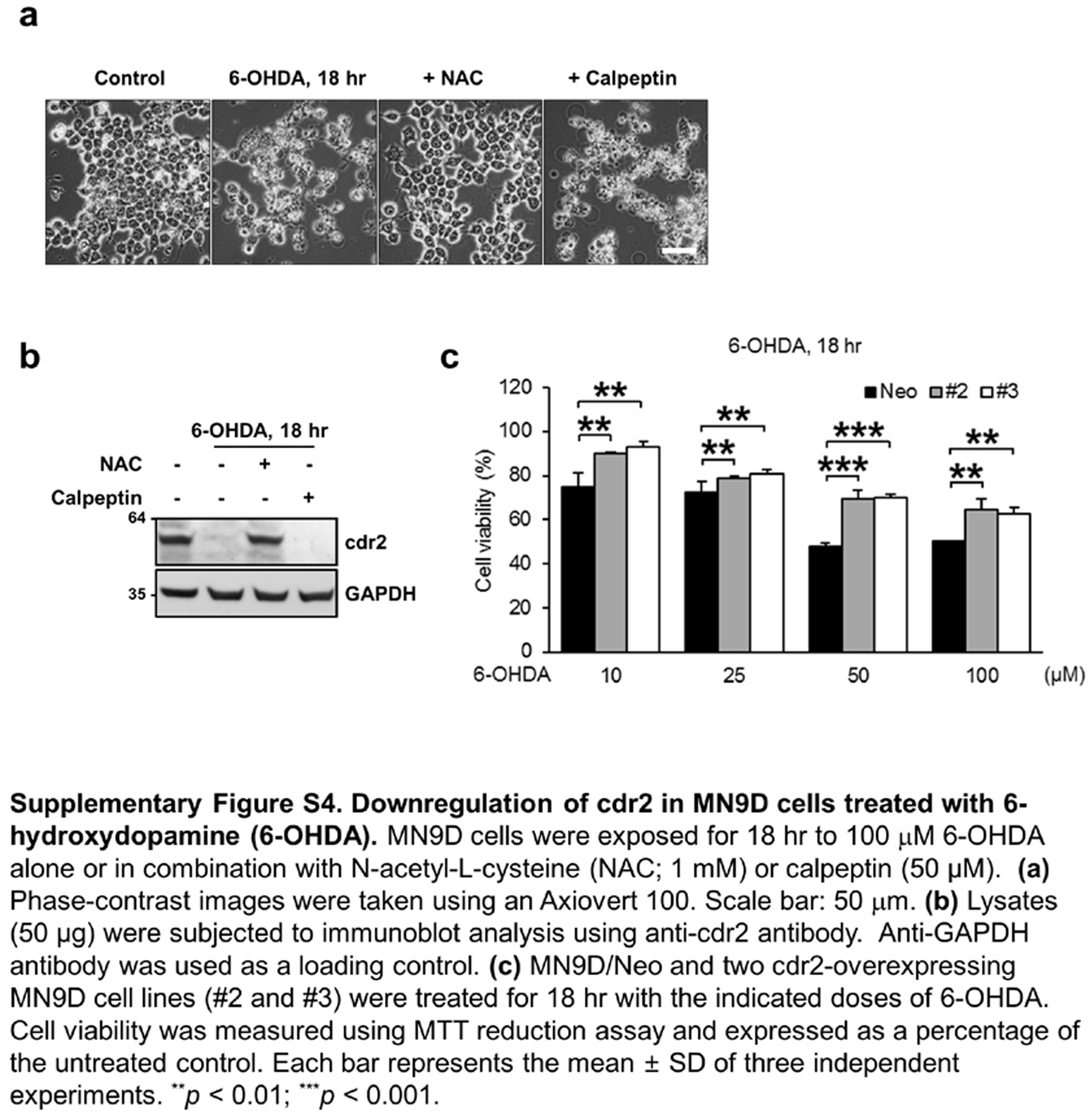

Supplement: Supplementary Figure 4 [file cddis2016151x4.tif]

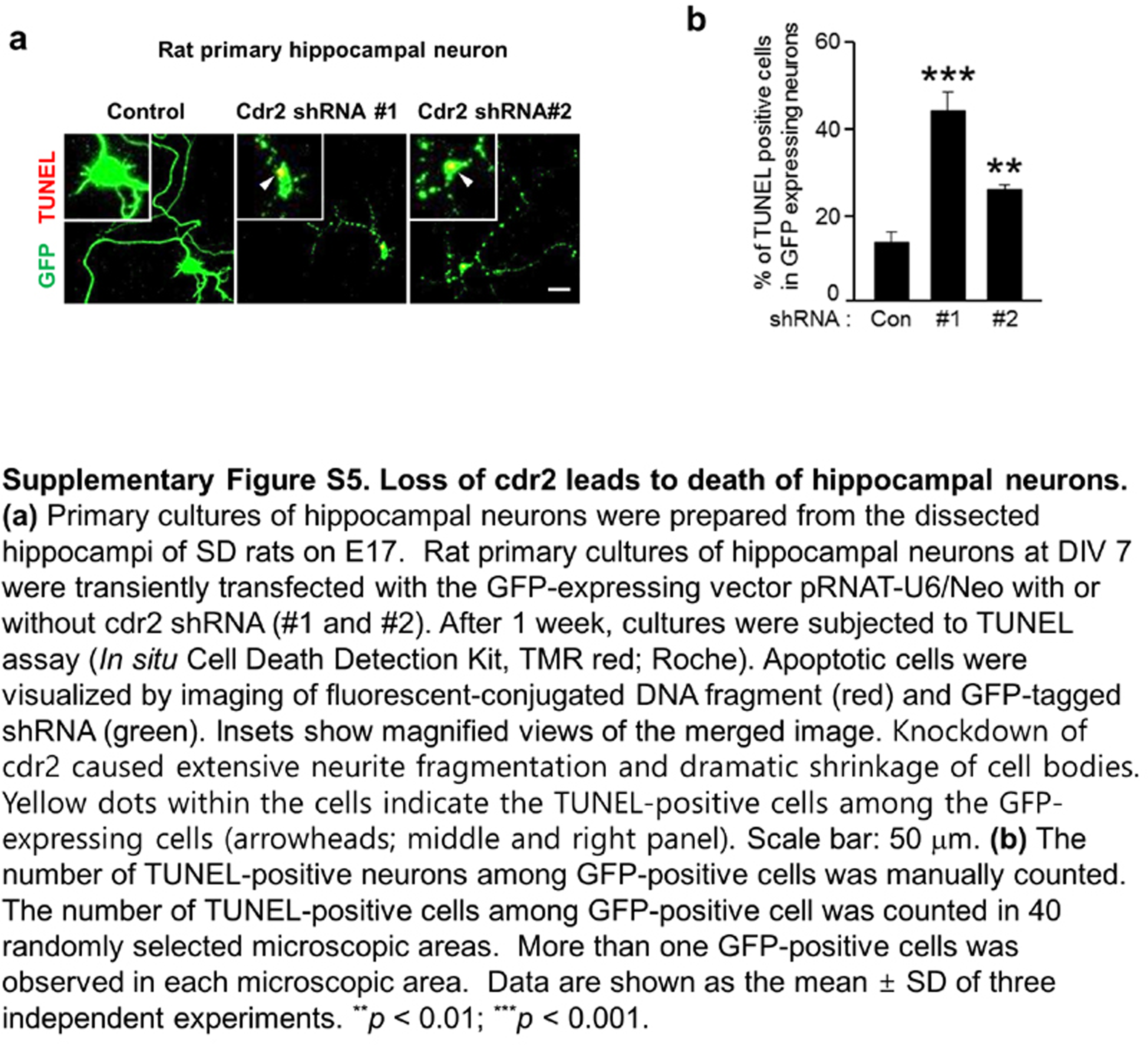

Supplement: Supplementary Figure 5 [file cddis2016151x5.tif]

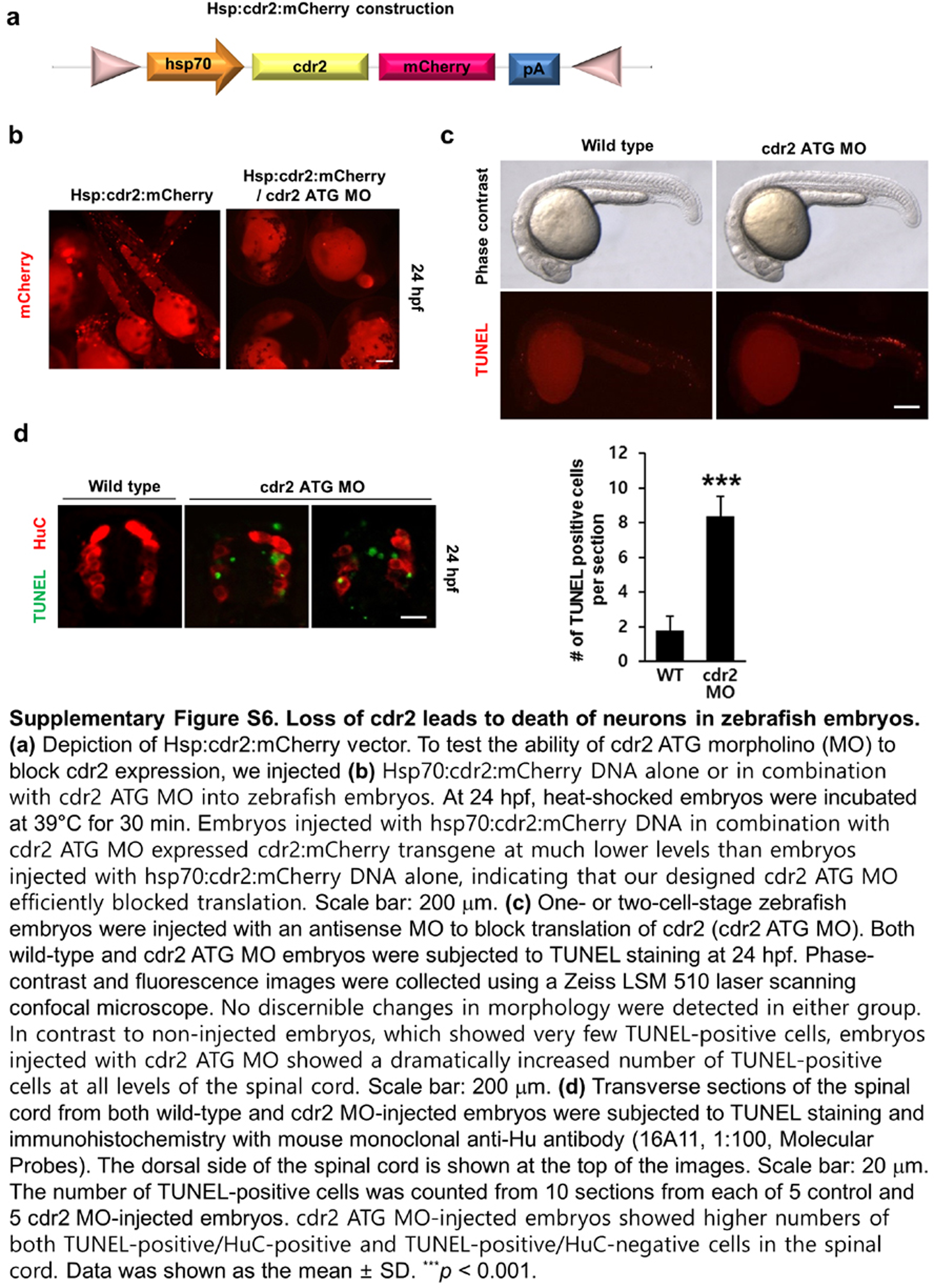

Supplement: Supplementary Figure 6 [file cddis2016151x6.tif]
